# Impact of baseline HIV-1 tropism on viral response and CD4 gains in antiretroviral-naïve patients

**DOI:** 10.1186/1758-2652-13-S4-O10

**Published:** 2010-11-08

**Authors:** E Seclén, M Gonzalez, L Martín-Carbonero, H Gellermann, V Cairns, M Distel, W Kadus, V Soriano, E Poveda

**Affiliations:** 1 Hospital Carlos III, Department of Infectious Diseases, Madrid, Spain; 2 Boehringer-Ingelheim, Ingelheim, Germany

## Purpose

To evaluate the influence of HIV-1 tropism on virologic and immunologic responses in antiretroviral-naïve HIV-1-infected patients enrolled in the ArTEN trial (atazanavir/r vs. nevirapine along with tenofovir/emtricitabine).

## Methods

Baseline plasma samples from patients enrolled in the ArTEN trial were tested genotypically for HIV tropism using geno2pheno (5.75% FPR) and PSSM (enhanced for X4 detection; Poveda et al, JAC 2009). Univariate and multivariate analyses were performed to find variables associated with virologic/immunological outcome. Parameters examined included gender, Hepatitis coinfection, HIV subtype, treatment arm, viral tropism, baseline CD4 counts and VL.

## Results

428 out of 569 randomized and treated patients could be analyzed, 146 on ATZ/r and 282 on NVP. Overall, 332 (76.9%) subjects were infected with subtype B variants and 45 (10.4%) were HCV coinfected. The V3 successful amplification rate was 92.1% (394/428). X4 variants were found in 14% (55/394) by geno2pheno and 29.2% (115/394) by PSSM, with no significant differences between treatment arms or HIV clade. See Table 1.

**Table 1:** 

Endpoint	HIV tropism (g2p)				Treatment arm			
	R5	X4	p(uni)	p(multi)	ATZ/r	NVP	p(uni)	p(multi)

Week 24								

% of patients with VL<50 copies/mL	83.2	60.9	0.001	0.012	77.2	82.1	0.281	0.384

CD4 gain (cells/mm^3^)	116[56-197]	117[66-172]	0.979	0.439	116[72-203]	111[45-111]	0.316	0.173

Week 48								

% of patients with VL<50 copies/mL	91.6	76.9	0.009	0.061	88.5	92.0	0.340	0.434

CD4 gain (cells/mm^3^)	156[83-244]	180[86-235]	0.729	0.616	180[99-251]	152[78-230]	0.037	0.008

At baseline, patients with X4 viruses by geno2pheno had higher VL (5.4 [IQR:5-5.7] vs. 5.2 [4.7-5.6] log copies/mL, p=0.044) and lower CD4 counts (145 [62-200] vs. 188 [134-260] cells/mm3, p<0.001) than those with R5 viruses. At weeks 24 and 48, the proportion of patients with VL <50 copies/mL was similar in both treatment arms, but lower in those with X4 than R5 viruses. The multivariate analysis confirmed HIV tropism as independent predictor of virologic response at week 24, along with baseline VL and CD4 count. In contrast, the extent of CD4 gains was not significantly determined by HIV tropism, but it was by baseline VL, CD4 count and treatment arm.

## Conclusions

HIV-1 tropism is an independent predictor of virologic response at week 24 in ARV-naïve patients treated with NVP or ATZ/r plus TDF/FTC. In contrast, CD4 gains are not determined by viral tropism. This observation may have important clinical implications, as it may be worthwhile testing for viral tropism, besides VL, CD4 counts and resistance before beginning any HAART regimen.
